# Genomic Signatures of Distributive Conjugal Transfer among Mycobacteria

**DOI:** 10.1093/gbe/evu175

**Published:** 2014-08-30

**Authors:** Tatum D. Mortimer, Caitlin S. Pepperell

**Affiliations:** ^1^Department of Medical Microbiology and Immunology, University of Wisconsin–Madison; ^2^Microbiology Doctoral Training Program, University of Wisconsin–Madison; ^3^Department of Medicine, Division of Infectious Diseases, University of Wisconsin–Madison

**Keywords:** lateral gene transfer, mycobacteria, *Mycobacterium canettii*, evolution

## Abstract

Distributive conjugal transfer (DCT) is a newly described mechanism of lateral gene transfer (LGT) that results in a mosaic transconjugant structure, similar to the products of meiosis. We have tested popular LGT detection methods on whole-genome sequence data from experimental DCT transconjugants and used the best performing methods to compare genomic signatures of DCT with those of LGT through natural transformation, conjugative plasmids, and mobile genetic elements (MGE). We found that DCT results in transfer of larger chromosomal segments, that these segments are distributed more broadly around the chromosome, and that a greater proportion of the chromosome is affected by DCT than by other mechanisms of LGT. We used the best performing methods to characterize LGT in *Mycobacterium canettii*, the mycobacterial species most closely related to *Mycobacterium tuberculosis*. Patterns of LGT among *M. canettii* were highly distinctive. Gene flow appeared unidirectional, from lineages with minimal evidence of LGT to isolates with a substantial proportion (6–13%) of sites identified as recombinant. Among *M. canettii* isolates with evidence of LGT, recombinant fragments were larger and more evenly distributed relative to bacteria that undergo LGT through natural transformation, conjugative plasmids, and MGE. Spatial bias in *M. canettii* was also unusual in that patterns of recombinant fragment sharing mirrored overall phylogenetic structure. Based on the proportion of recombinant sites, the size of recombinant fragments, their spatial distribution and lack of association with MGE, as well as unidirectionality of DNA transfer, we conclude that DCT is the predominant mechanism of LGT among *M. canettii*.

## Introduction

Lateral gene transfer (LGT) is a driving force in the adaptation of bacterial populations, introducing novel genetic material, shuffling combinations of adaptive mutations, and discarding deleterious mutations that accumulate during asexual reproduction (Gogarten and Townsend 2005; Takeuchi et al. 2014). LGT in bacteria can occur by transformation, transduction, or conjugation, and after uptake, DNA is incorporated into the recipient chromosome through homologous or nonhomologous recombination (Thomas and Nielsen 2005).

Little is known about LGT among mycobacteria, despite their importance as pathogens and abundance in the environment. It has been hypothesized that virulence factors in *Mycobacterium tuberculosis, Mycobacterium abscessus,* and *Mycobacterium avium* were acquired by LGT from other species of bacteria (Marri et al. 2006; Ripoll et al. 2009; Veyrier et al. 2009)*.* LGT among *M. avium* has also been reported (Krzywinska et al. 2004). Extant populations of *M. tuberculosis* appear to evolve clonally (Supply et al. 2003; Comas et al. 2013; Pepperell et al. 2013). However, the extent to which natural populations of other mycobacteria engage in LGT and potential mechanism(s) of transfer among mycobacteria are unexplored. Deciphering patterns of LGT among mycobacteria is vital for understanding evolution of pathogenic and nonpathogenic members of this genus.

*Mycobacterium canettii* (also called smooth tubercle bacilli) is a close relative of *M. tuberculosis* that has been isolated on rare occasions from cases of tuberculosis in East Africa. In contrast to *M. tuberculosis,* genetic data from *M. canettii* have clear evidence of intergenomic recombination (Gutierrez et al. 2005). A recent analysis of whole-genome sequence (WGS) data from nine isolates of *M. canettii* found statistical support for recombination in the alignment with a significant pairwise homoplasy index (PHI) test (Bruen et al. 2006) and regions with unusually high densities of single nucleotide polymorphisms (SNP) (Supply et al. 2013). More detailed characterization of LGT among *M. canettii* would provide insight into the evolution of this interesting organism. *Mycobacterium canettii* is the mycobacterial species most closely related to *M. tuberculosis* complex (Brosch et al. 2002), and a better understanding of its evolution would also contribute to our understanding of the emergence of human-pathogenic mycobacteria.

Distributive conjugal transfer (DCT) is a newly discovered mechanism of bacterial conjugation described in *Mycobacterium smegmatis*. Multiple, noncontiguous tracts of chromosomal DNA are transferred from a donor to recipient cell during DCT, and transconjugant progeny are mosaics of the parental genomes (Gray et al. 2013). DCT-transferred tracts are large, with an average size of 33 kb. Homologous recombination is required in the recipient cell, suggesting that this mechanism of LGT occurs primarily among closely related bacteria (Wang et al. 2003). DCT in *M. smegmatis* is regulated by the type VII secretion system ESX-1 and requires the lipoprotein-metalloproteinase LpqM, both of which are conserved across mycobacterial species (Coros et al. 2008; Nguyen et al. 2009).

The mosaicism produced by DCT sets it apart from other mechanisms of bacterial LGT, and existing methods of identifying bacterial recombination may not be optimal for this mechanism of exchange. In this study, we tested several recombination detection methods previously used on bacterial sequence data for their ability to identify DCT using WGSs from well-characterized *M. smegmatis* experimental transconjugants. We compared the genomic signatures of DCT with other mechanisms of LGT including natural transformation, MGEs, and conjugative plasmids. Additionally, we used the best performing method to identify the likely mechanism of LGT among *M. canettii*.

## Materials and Methods

### Data Set

We analyzed WGS data from *M. smegmatis* transconjugants described in Gray et al. (2013) and extant isolates of *M. canettii* (Supply et al. 2013)*.* Accession numbers for WGS data from *M. smegmatis* and *M. canettii* are listed in supplementary table S1, Supplementary Material online. We also compared our results with BRATNextGen analyses of WGS from *Streptococcus pneumoniae*, described in Marttinen et al. (2012), *Staphylococcus aureus*, described in Castillo-Ramírez et al. (2012), and *Enterococcus faecium,* described in de Been et al. (2013). Global data sets of *Str. pneumoniae* and *Sta. aureus* are described in supplementary table S1, Supplementary Material online.

### Assembly of *M. smegmatis* Transconjugant Genomes

Before assembly, read data were quality trimmed to a minimum quality score of 15 using TrimGalore (Felix Krueger, Trim Galore!, http://www.bioinformatics.babraham.ac.uk/projects/trim_galore/, 2013) and reduced to a uniform coverage using digital normalization (Brown CT, Howe A, Zhang Q, Pyrkosz AB, Brom TH, unpublished data, which were downloaded from http://arxiv.org/abs/1203.4802, last accessed September 2, 2013). De novo assembly of *M. smegmatis* paired-end data with read length of 100 bp was done with MaSuRCA (Zimin et al. 2013). For read lengths of 50 bp, we used BWA to map reads to the donor strain mc^2^155 (Li and Durbin 2009). BWA-MEM was used to map reads of length 75 bp (Li H, unpublished data, which were downloaded from http://arxiv.org/abs/1303.3997, last accessed September 2, 2013). We used Picard (Picard, http://picard.sourceforge.net, 2013) to add read group information and mark duplicates, and GATK for indel realigning and SNP calling (DePristo et al. 2011).

### Whole Genome and SNP Alignments

We used Kodon (v 3.62, Applied Maths) for WGS alignment and SNP identification. De novo assemblies of *M. smegmatis* were aligned to the reference strain mc^2^155. WGSs of *M. canettii* were aligned to CIPT 140010059 (STB-A). We used in-house Python scripts to create SNP alignments from Variant Call Format (VCF) files produced by GATK in reference-guided assemblies of *M. smegmatis*. Before downstream analysis, repetitive regions, including PE/PPE genes, phage, and transposable elements, and regions with missing data were removed from the alignment.

### Recombination Detection Methods

We used *M. smegmatis* transconjugant data to evaluate the accuracy of recombination detection methods in identifying and characterizing recombinant fragments resulting from DCT. We used the pairwise homoplasy index (PHI), as implemented in SplitsTree4, to detect the presence of recombination in whole-genome alignments and SNP alignments of *M. smegmatis* (Bruen et al. 2006; Huson and Bryant 2006)*.* We used the pairwise program within LDhat Version 2.2 (Auton and McVean 2007) to estimate the population recombination rate from SNP alignment data. cBrother (Minin et al. 2005; Fang et al. 2007) was run with default settings using donor and recipient strains as representative sequences and transconjugants as query sequences. We used progressiveMauve (Darling et al. 2010) to produce whole-genome alignments of *M. smegmatis* for input into ClonalFrame (Didelot and Falush 2007). The ClonalFrame Markov chain Monte Carlo had a total length of 150,000 iterations with 50,000 burn-in iterations. Three independent runs of ClonalFrame were compared for convergence of results. ClonalOrigin was run according to instructions here: https://code.google.com/p/clonalorigin/wiki/FromGenomeAssemblyToRecombination (last accessed September 18, 2013). We used BRATNextGen to analyze whole-genome alignments of *M. smegmatis* de novo assemblies and SNP alignments of *M. smegmatis* reference-guided assemblies. One hundred permutations of the analysis were used to calculate the significance of detected recombinant regions (*P* < 0.05) as in other studies using BRATNextGen (Castillo-Ramírez et al. 2012; Marttinen et al. 2012; de Been et al. 2013).

We assessed the accuracy of recombination detection methods by comparing known breakpoints in experimental data with breakpoints output by each program. We used the highest resolution *M. smegmatis* data set for this analysis, corresponding to transconjugants with sufficient coverage to allow de novo assembly. We used a threshold site-specific recombination probability of 0.9 to create recombination breakpoints from ClonalFrame and ClonalOrigin output. The positive predictive value (PPV) of each method was defined as the ratio of the number of positions correctly identified as recombinant to the total number of positions identified as recombinant. The negative predictive value (NPV) was defined as the ratio of the number of positions correctly identified as nonrecombinant to the total number of positions identified as nonrecombinant.

To close incorrect gaps in the BRATNextGen analysis of *M. smegmatis*, we combined regions spaced less than 475 bp apart, which closes less than 5% of correct gaps from the true breakpoint data (supplementary fig. S2, Supplementary Material online). We also used BRATNextGen software to detect recombination using an SNP alignment of *M. canettii, Str. pneumoniae,* and *Sta. aureus* genomes with the same settings as previously used with *M. smegmatis* transconjugants. In order to allow comparisons across bacteria, we applied the gap correction to BRATNextGen output from all species included in our sample.

### Analyses of Spatial Bias

We divided the WGS alignment of each species into sliding windows of 100 kb, and counted the number of recombination events within each window. We also compared pairs of strains in the data sets to identify overlapping recombinant regions and calculate the proportion of recombinant fragments shared between each pair. We compared the observed data to a null distribution created by randomly placing recombinant fragments of equal number and size to the observed data and repeating the sliding window and shared regions analyses. The sliding window and overlapping regions analyses were simulated 1,000 times.

### Phylogenetic Analysis of *M. canettii* Recombinant Regions

We aligned recombinant fragments (defined by BRATNextGen) to the complete sample of *M. canettii* sequences. We used MrBayes 3.2.1 to infer phylogenies from these alignments (Ronquist and Huelsenbeck 2003). We used genetic distances between bacterial isolates on these phylogenies to find the most closely related nonrecombinant sequence in the sample and putative origin of the fragment. We also examined recombinant regions unique to each *M. canettii* strain. Using BLASTn, we queried unique sequence greater than 1,000 bp in length against the nucleotide collection (nr/nt) and whole-genome shotgun contigs (wgs) databases.

## Results

### Method Accuracy

In order to identify the LGT detection methods capable of detecting and characterizing DCT among mycobacteria, we analyzed an alignment of WGS from experimental *M. smegmatis* transconjugants with previously defined breakpoints (table 1). The PHI test identified recombination in the *M. smegmatis* alignment with high statistical confidence (*P* = 0.0). PHI does not provide the location of putative recombination breakpoints, nor does it estimate the rate of recombination. We used LDhat to generate maximum-likelihood estimates of the rate of recombination among *M. smegmatis*. The distribution of likelihoods estimated with LDhat was flat over a broad range of recombination rates (supplementary fig. S1, Supplementary Material online). cBrother did not detect any recombination within the *M. smegmatis* transconjugant genomes tested. Given these results, we did not evaluate LDhat and cBrother any further.

**Table 1:** Recombination Detection Methods Tested

Method	Version	Reference
BRATNextGen	—	Marttinen et al. (2012)
cBrother	2.0	Minin et al. (2005); Fang et al. (2007)
ClonalFrame	1.2	Didelot and Falush (2007)
ClonalOrigin	—	Didelot et al. (2010)
GENECONV	1.81	Sawyer (1989)
LDhat	2.2	Auton and McVean (2007)
PHI in SplitsTree	4.12.6	Bruen et al. (2006); Huson and Bryant (2006)

The output from analyses using BRATNextGen and GENECONV consists of estimated recombination breakpoints. ClonalFrame estimates the probability of recombination versus mutation at each polymorphic site in the alignment. ClonalOrigin infers the probability of recombination across the genome and also models the origins of recombination events. We compared outputs from analyses of *M. smegmatis* alignments with the known breakpoints to calculate PPV and NPV for each method (table 2)*.* In addition to recombination probabilities for each polymorphic site, ClonalFrame estimates the distribution of recombinant fragment lengths. The mean of this exponential distribution was 642 bp, lower than the experimentally defined mean of 33 kb for these genomes (Gray et al. 2013). GENECONV and ClonalOrigin can be used to estimate the origins of recombinant sequence. However, given the inaccuracy of these methods in identifying recombinant fragments, we did not evaluate their accuracy in determining the origin of these fragments.

**Table 2:** Accuracy of Recombination Detection Methods

Method	PPV^a^	NPV^b^
BRATNextGen	0.86	0.99
GENECONV	0.35	0.99
ClonalFrame	0.57	0.94
ClonalOrigin	0.50	0.97

^a^No. of true recombinant site/(No. of true recombinant sites + No. of false recombinant site).

^b^No. of true nonrecombinant sites/(No. of true nonrecombinant sites + No. of false nonrecombinant sites).

**Fig. 1.—:**
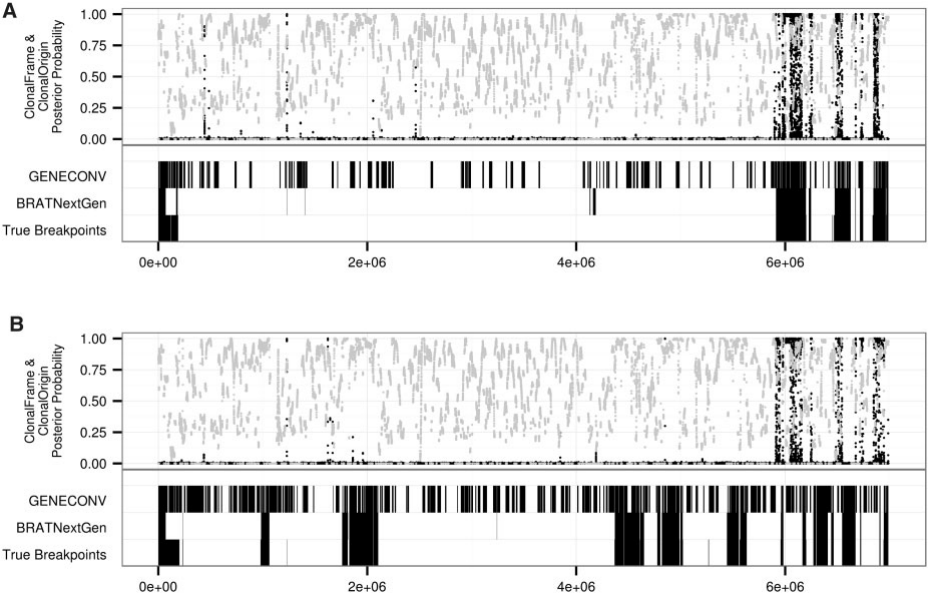
Results of detection methods on known transconjugants. Recombination breakpoints from BRATNextGen and GENECONV and posterior probability of recombination from ClonalFrame (black dots) and ClonalOrigin (gray dots) are shown for *M. smegmatis* transconjugants, Km6.9a (*A*) and Km4.5a (*B*). True breakpoints for recombinant tracts in Km6.9a and Km4.5a are from Gray et al. (2013). BRATNextGen performed best at identifying recombinant regions transferred through DCT in *M. smegmatis*.

BRATNextGen was the most accurate of these methods of identifying recombination breakpoints (table 2 and fig. 1). BRATNextGen also outputs putative origins within the sample for recombinant tracts. The origin of transferred sequence was identified correctly in only 69 of 116 cases (59.5%). In comparing the BRATNextGen LGT fragments with experimentally defined breakpoints, we also found that the method divided some large recombinant fragments into multiple smaller fragments. We examined the size distributions of gaps between experimentally defined recombination breakpoints and incorrect gaps from BRATNextGen output (supplementary fig. S2, Supplementary Material online). We applied a gap closure correction that joins fragments less than 475 bp apart, corresponding to closure of fewer than 5% of true gaps between *M. smegmatis* recombinant fragments. After closing small gaps between recombinant fragments, the size distribution of fragments identified by BRATNextGen was closer to the true size distribution for *M. smegmatis* (supplementary fig. S3, Supplementary Material online).

### Genomic Signatures of LGT in Different Bacterial Species

In addition to identifying best performing methods of identifying DCT from WGS, we compared genomic signatures of DCT with those of LGT in other bacterial species. We compared the *M. smegmatis* transconjugant data with published BRATNextGen analyses of WGS from *Str. pneumoniae, Sta. aureus,* and *E. faecium*. The published data include examples of natural transformation, LGT through mobile genetic elements (MGE), and transfer through conjugative plasmids. We also used BRATNextGen to characterize recombination in *M. canettii*, the sister species to *M. tuberculosis*, which is distantly related to *M. smegmatis*.

**Fig. 2.—:**
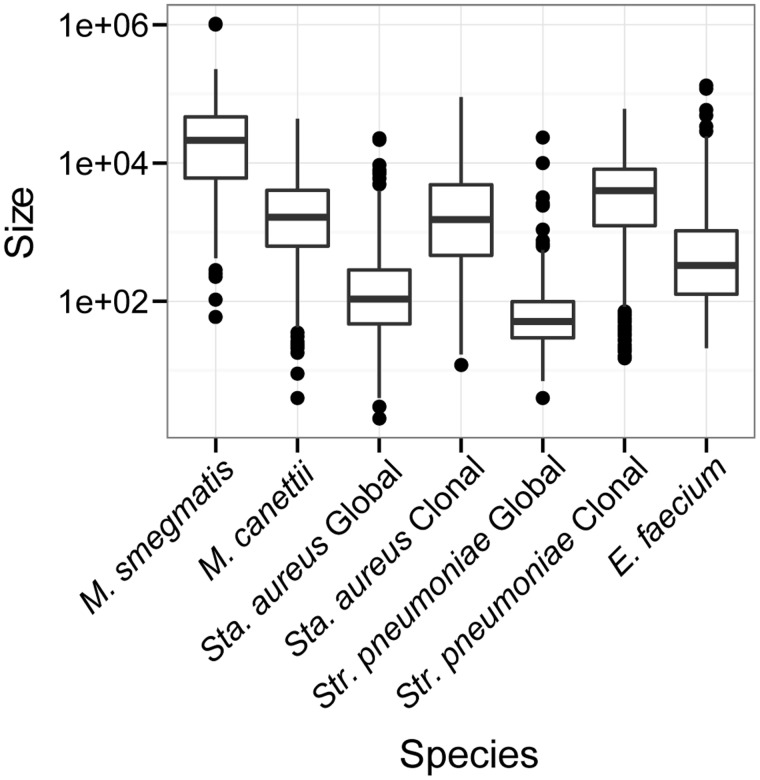
Size distributions of recombinant fragments. Boxplot of LGT fragment sizes in several species of bacteria. LGT fragments identified using BRATNextGen except for *M. smegmatis*, which shows the size distribution of recombinant fragments identified by Gray et al. (2013). Recombinant fragments identified with BRATNextGen were combined if the gap between them was less than 475 bp (supplementary figs. S2 and S3, Supplementary Material online). Size shown in base pairs on the *y* axis is on a log scale. We observed an effect of sampling on the size distribution of LGT fragments: Fragment sizes were smaller in diverse samples of *Sta. aureus* and *Str. pneumoniae* relative to samples taken from a single lineage. LGT fragments in the diverse sample of *M. canettii* are larger than those found in comparable samples of other bacteria. Samples of *M. smegmatis* were taken immediately after experimental mating: We hypothesize that in natural populations the large LGT fragments observed here would be interrupted (and shortened) by subsequent mutation and LGT.

In order to facilitate direct comparisons with BRATNextGen output from the experimental transconjugant data, we used the same gap closing correction that was used for *M. smegmatis*. Comparison of the size distributions of recombinant fragments shows that DCT among *M. smegmatis* is characterized by transfer of larger sized fragments relative to LGT among other species of bacteria (fig. 2). The size of recombinant fragments detected by BRATNextGen in *M. canettii* ranged from 4 to 43,710 bp (fig. 2) with a mean of 3.3 kb.

In addition to transfer of relatively large fragments, DCT is characterized by broad effects on the *M. smegmatis* genome, with a greater proportion of sites affected by recombination than *Sta. aureus*, *Str. pneumoniae,* and *E. faecium* (supplementary table S2, Supplementary Material online). Similar to *M. smegmatis*, for which the range was 1–25%, between 6% and 13% of the genome sequence of seven *M. canettii* isolates was identified as recombinant. An additional two isolates—STB-J and STB-K—have little evidence of recombination (0.02–0.06% recombinant sites).

### Bias in Location of Transferred Fragments

**Fig. 3.—:**
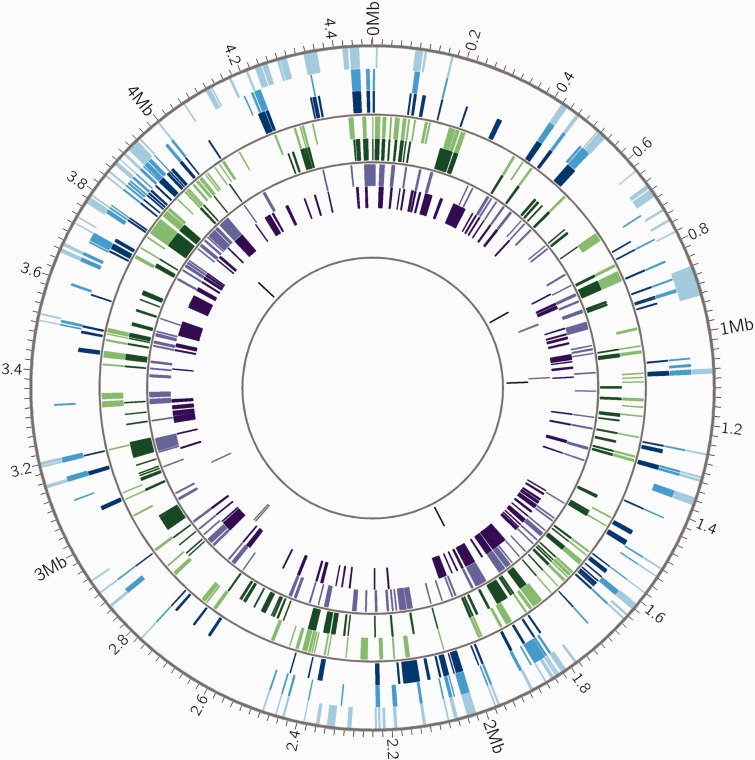
Distribution of recombinant fragments across *M. canettii* chromosomes. *Mycobacterium canettii* recombinant fragments identified by BRATNextGen are shown as colored blocks. Genomic positions are in reference to *M. canettii* STB-A (CIPT 140010059). *Mycobacterium canettii* strain identifiers in order from outermost circle to innermost circle are STB-A (light blue), STB-D (medium blue), STB-E (dark blue), STB-L (light green), STB-G (dark green), STB-I (light purple), STB-H (dark purple), STB-K (gray), STB-J (black). Thin gray circles divide genomes into groups defined by phylogenetic analysis (supplementary fig. S5, Supplementary Material online). Prior to identification of recombinant fragments, regions prone to homoplasy such as PE/PPE genes and transposons were removed from the alignment. Plot made with Circos (Krzywinski et al. 2009). Recombinant DNA sequences are shared by closely related strains of *M. canettii*, which suggests that they are maintained in situ by clonal evolution following LGT events. This pattern is distinct from hot spots (see supplementary fig. S4, Supplementary Material online), which are shared across all strains. The frequency of recombination appears to vary among bacterial isolates, with two of the nine isolates (STB-K and STB-J) exhibiting little evidence of LGT.

In order to investigate bias in the location of recombinant fragments, we divided genomes into windows of 100 kb and calculated the number of recombination events for each window. We find for all bacterial species in our sample that there are more windows with no recombination events than expected if the fragments were placed at randomly selected locations throughout the genome. This suggests that there are genomic regions in which transferred fragments of DNA are infrequently inserted due to impacts on fitness, structural barriers to recombination or other reasons (i.e., recombination “cold spots”) (table 3). We also looked for evidence of recombination “hot spots.” We performed pairwise comparisons of all strains in each data set and calculated the proportion of recombinant regions that were shared. In the presence of hot spots, we expect strains to have more overlapping areas of recombination than observed when fragments are randomly placed. We found evidence of hot spots in all species, relative to a null distribution in which recombinant fragments were placed at randomly chosen locations (table 4). This positive spatial bias was less marked in *M. canettii* and *M. smegmatis* than in the other species of bacteria. LGT hot spots in *Str. pneumoniae*, *Sta. aureus*, and *E. faecium* are easily observed when recombinant fragments are plotted; they are less evident in data from *M. smegmatis* and *M. canettii* (fig. 3 and supplementary fig. S3, Supplementary Material online; Gray et al. 2013). In *M. canettii*, overlapping fragments tend to be shared between subsets of isolates that cluster together on the phylogeny rather than being shared across all strains.

**Table 3:** Proportion of 100-kb Windows with No Recombination Events: Observed and Simulated, Randomly Placed Fragments

Species	Observed (Mean)^a^	Simulated (Mean)^b^	*P* Value^c^
*Mycobacterium smegmatis*	0.93	0.90	<*0.001*
*Mycobacterium canettii*	0.31	0.25	<*0.001*
*Streptococcus pneumoniae*	0.75	0.73	<*0.001*
*Staphylococcus aureus*	0.86	0.83	<*0.001*
*Enterococcus faecium*	0.54	0.42	<*0.001*

^a^Mean proportion of windows with no recombination events across strains.

^b^Recombinant fragments equal in size and number to observed data were randomly distributed across the genome and proportion of windows with no recombination events was calculated. These simulations were repeated 1,000 times.

^c^A *P* value was calculated by comparing the observed proportion of windows with no recombination to the distribution of simulations (*P* = proportion of simulated distribution ≥ observed). Significant *P* values are given in italics.

**Table 4:** Proportion of Pairwise Comparisons with Overlapping Recombinant Fragments: Observed and Simulated, Randomly Placed Fragments

	25–49% Overlapping			50–74% Overlapping			>75% Overlapping		
Species	Observed	Mean of Simulations^a^	*P* Value^b^	Observed	Mean of Simulations^a^	*P* Value^b^	Observed	Mean of Simulations^a^	*P* Value^b^
*Mycobacterium smegmatis*	0.06	0.016	*0.024*	0.0	3.3 × 10^−5^	1.0	0.0	0.0	1.0
*Mycobacterium canettii*	0.14	0.0	<*0.001*	0.06	0.0	<*0.001*	0.0	0.0	1.0
*Streptococcus pneumoniae*	0.07	0.004	<*0.001*	0.02	9.1 × 10^−5^	<*0.001*	0.04	1.2 × 10^−6^	<*0.001*
*Staphylococcus aureus*	0.06	0.002	<*0.001*	0.08	0.001	<*0.001*	0.09	6.8 × 10^−5^	<*0.001*
*Enterococcus faecium*	0.12	0.005	<*0.001*	0.08	0.0001	<*0.001*	0.07	2.8 × 10^−6^	<*0.001*

^a^Recombinant fragments equal in size and number to observed data were randomly distributed across the genome and the proportion of overlapping recombinant regions was calculated between each simulated genome. These simulations were repeated 1,000 times.

^b^A *P* value was calculated by comparing the observed proportion of pairwise comparisons to the distribution of proportions from the simulations (*P* = proportion of simulated distribution ≥ observed). Significant *P* values are given in italics.

### Characteristic Features of LGT in *M. canettii*

Unlike *Sta. aureus* and *Str. pneumoniae*, LGT in our sample of *M. canettii* does not appear to have been mediated by phage or other types of MGE. Using the annotation of STB-A, we examined the content of recombinant fragments and found no fragments associated with phage or MGE. This is also what we observed with the *M. smegmatis* transconjugant data; none of the recombinant fragments was associated with phage or MGE.

As with *M. smegmatis* DCT transconjugants (Gray et al. 2013), *M. canettii* recombinant fragments are widely distributed across the genomes (fig. 3). Interestingly, strains that cluster together in the *M. canettii* phylogeny (shown in supplementary fig. S5, Supplementary Material online) share many recombinant tracts (fig. 3). This suggests that LGT-transferred segments are maintained in situ during subsequent clonal evolution. In contrast, we would expect fragments transferred through phage or MGE to be repeatedly gained and lost.

**Fig. 4.—:**
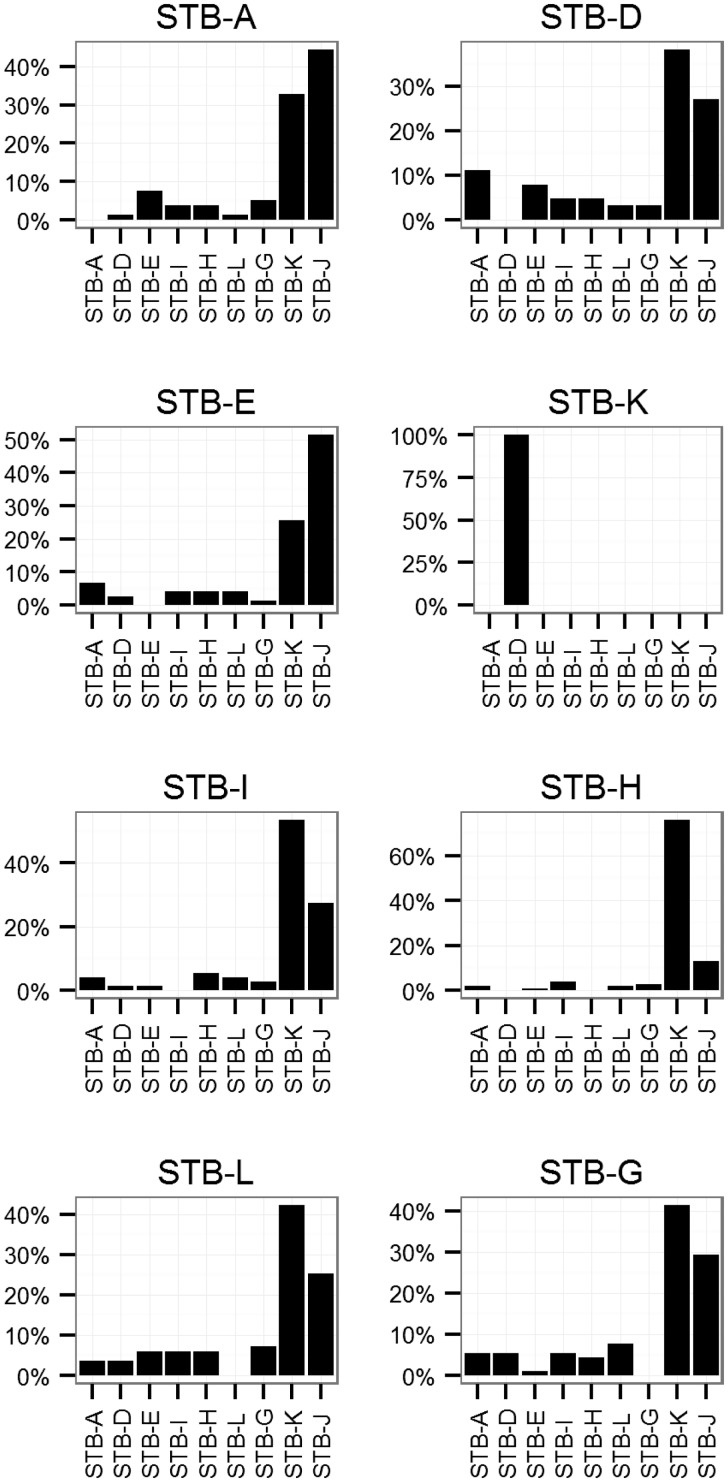
Origins of recombinant fragments in *M. canettii*. We used MrBayes v3.2.1 (Ronquist and Huelsenbeck 2003) to identify the most closely related strain of *M. canettii* for each recombinant fragment in the alignment. When the most closely related strain was also identified as recombinant in that location, the most closely related nonrecombinant strain was used instead. Each plot shows all the recombinant tracts greater than 1 kb in each strain. STB-J did not contain any recombinant tracts that met this threshold. STB-K only had one tract that met this threshold (858686–860855). Putative origins for recombinant tracts are shown on the *x* axis. The percentage of recombinant tracts with each origin is shown on the *y* axis. STB-K and STB-J are the most closely related strains for the majority (76%) of recombinant tracts.

Directionality of transfer could offer some further clues about the mechanism of LGT among *M. canettii*. We used a phylogenetic approach to identify the origins of recombinant fragments in *M. canettii* (none of the LGT detection methods performed well in this respect). After identifying portions of the alignment with recombination in at least one strain, we created Bayesian phylogenies of these fragments with MrBayes (Ronquist and Huelsenbeck 2003) and calculated the phylogenetic distance between each strain, assuming that recombinant fragments would be closest to the donor sequence. These analyses implicate STB-J and STB-K as sources of the majority (76%) of recombinant fragments in the other genomes (fig. 4). In order to investigate the origins of recombinant fragments that are not shared among all *M. canettii* (i.e., not found on the alignment), we searched for homologous sequences using BLAST (Altschul et al. 1990). We did not find any unique sequences in *M. canettii* shared with those in National Center for Biotechnology Information’s nr/nt or wgs databases beyond those shared with *M. tuberculosis* and the CRISPR-associated proteins identified previously (Supply et al. 2013).

## Discussion

DCT is a new paradigm for bacterial LGT. The amount of donor sequence incorporated into the recipient chromosome during one mating dwarfs that of other LGT mechanisms, and the mosaicism of transconjugants approaches that of eukaryotic meiosis (Gray et al. 2013). Many recombination detection methods have been developed to identify recombinant fragments in bacterial genomes. However, these programs have not been tested for their ability to detect LGT by DCT.

We evaluated DCT detection accuracy of a series of LGT detection methods using WGS data from experimental *M. smegmatis* transconjugants. We were not able to estimate a rate of recombination from these data with LDhat (Auton and McVean 2007); potential explanations include the average tract length of 100 bp used to test the gene conversion model (McVean et al. 2002), an inability to model hotspots for gene conversion (the recombination hotspots model is only available for crossing over), and our use of experimental rather than population level data. cBrother (Minin et al. 2005; Fang et al. 2007) failed to identify recombination in the sample. The dual multiple change point model implemented in cBrother has been used primarily for viral genomes or bacterial genes (Martinez-Garcia et al. 2012; Engel et al. 2013), and the model may not be appropriate for bacterial WGS with extensive recombination. The PHI test as implemented in SplitsTree4 (Bruen et al. 2006; Huson and Bryant 2006) correctly identified recombination in the *M. smegmatis* transconjugants. GENECONV (Sawyer 1989) and ClonalFrame (Didelot and Falush 2007) have been used previously to identify putative recombination in *M. tuberculosis* (Namouchi et al. 2012); we found that both of these programs had low accuracy in characterizing transfer between *M. smegmatis* strains through DCT (GENECONV PPV: 0.35, NPV: 0.99; ClonalFrame PPV: 0.57, NPV: 0.94). GENECONV compares pairs of sequences and identifies those that are unusually similar, which should detect recombination events within the provided sequences. Although GENECONV did detect the true events, it also detected many false positives throughout the genome (fig. 1). These may be regions where the donor and recipient genomes are more similar than average. ClonalFrame is designed to identify recombinant fragments from outside the sample, which may explain its poor performance on this data set (Didelot and Falush 2007). However, ClonalOrigin (PPV: 0.5, NPV: 0.97) was designed to detect recombinant fragments within the sample, and despite better performance on other bacterial WGS (Didelot et al. 2010), it did not improve on the results from ClonalFrame. BRATNextGen (Marttinen et al. 2012), based on a Bayesian change-point clustering model, correctly identified recombinant tracts in *M. smegmatis* and had fewer false positives than other methods (PPV: 0.86, NPV: 0.99). We did observe that BRATNextGen divided some long recombinant tracts into multiple smaller tracts. For future study of DCT among bacterial populations using WGS, we recommend the use of BRATNextGen over other currently available methods, possibly with a gap closure correction.

DCT in *M. smegmatis* has a profound impact on the recipient genome; donor sequence comprises up to 25% of the transconjugant genomes after just one mating. Tracts are dispersed throughout the genome, with as many as 33 tracts transferred at one time (Gray et al. 2013). We compared genomic signatures of *M. smegmatis* DCT with data from *Str. pneumoniae*, *E. faecium*, and *Sta. aureus.* Using the BRATNextGen analysis of WGS from *M. smegmatis* and these other bacteria, we have confirmed and further characterized the hallmarks of DCT.

DCT in *M. smegmatis* is controlled by a type VII secretion system (ESX-1) and a lipoprotein-metalloproteinase, LpqM (Coros et al. 2008; Nguyen et al. 2009). These genes are not present in *Str. pneumoniae*, *Sta. aureus*, or *E. faecium*. *Streptococcus pneumoniae* is naturally transformable and also undergoes LGT through phage and conjugative elements (Croucher et al. 2011), whereas LGT in *Sta. aureus* is thought to be mediated primarily by phage and the SCC*mec* MGE (Castillo-Ramírez et al. 2012). The described mechanisms of LGT among *E. faecium* are through conjugative plasmids and phage (Willems et al. 2012; de Been et al. 2013; Lebreton et al. 2013).

DCT-transferred fragments in *M. smegmatis* were much larger than recombinant fragments in other bacteria (fig. 2), suggesting that fragments transferred by phage, MGE, and plasmids are smaller than those transferred through DCT. We also found evidence of a sampling effect on the size distribution of recombinant fragments. We analyzed data from extant clones of *Str. pneumoniae* and *Sta. aureus* (Croucher et al. 2011; Castillo-Ramírez et al. 2012) as well as from globally diverse samples of these bacteria (see *Methods*). LGT fragments identified in genetically diverse, globally extant samples of *Sta. aureus* and *Str. pneumoniae* were smaller relative to the samples of individual clones (fig. 2). We hypothesize that this is because the clonal samples have diverged over a shorter period of time, and there have thus been fewer opportunities for subsequent mutations to disrupt the recombinant segments. Our *M. smegmatis* data set consists of transconjugants sequenced soon after mating; the effects of DCT on genetic variation in natural populations of *M. smegmatis* are not known. We hypothesize that in diverse samples of natural *M. smegmatis* populations, the large DNA sequence fragments transferred through DCT are likely to show evidence of disruption by subsequent mutations and overlapping LGT events.

The size distribution of recombinant fragments produced by BRATNextGen in this *Str. pneumoniae* data set is consistent with fragments identified using a different method in two *Str. pneumoniae* clones, including the one analyzed here (Mostowy et al. 2014). This consistency provides further support for the accuracy of BRATNextGen in identifying and characterizing bacterial LGT.

In addition to the generally larger size of DCT-transferred fragments, the proportion of the recipient genome replaced with donor sequence is greater in *M. smegmatis* transconjugants than other organisms (supplementary table S2, Supplementary Material online).

Based on our analyses of bias in the location of recombinant fragments, all species examined appear to have “cold spots” where recombination does not occur. DCT requires homologous recombination (Wang et al. 2003), and cold spots in the *M. smegmatis* alignment could represent regions that are too divergent between the donor and recipient genomes to allow recombination. Cold spots could also be under strong functional constraint, such that recombination is not tolerated (Nakamura et al. 2004). This explanation would be consistent with other analyses of bacterial LGT and eukaryotic meiosis (Petes 2001; Yahara et al. 2012). Further study of cold spot regions may point to a mechanism of recombination suppression in bacterial genomes.

Recombination “hot spots” have been described for natural transformation, conjugative plasmid, and MGE-mediated transfer in *Str. pneumoniae*, *E. faecium*, and *Sta. aureus* (Croucher et al. 2011; Castillo-Ramírez et al. 2012; de Been et al. 2013)*.* Our results confirm these findings. We also find evidence of some positive bias in the location of DCT-transferred fragments in *M. smegmatis*, but this bias is weaker than for comparator bacteria. *Mycobacterium smegmatis* hot spots could be driven by the locations of the antibiotic markers used for selection of transconjugants or multiple origins of transfer (*bom* sites) around the genome (Wang et al. 2005). Alternatively, both hot spots and cold spots could arise from a tendency for recombinant fragments to colocalize.

A greater sample size of *M. smegmatis* transconjugant genomes would be useful to determine whether or not hot spots and cold spots appear in stable locations (e.g., due to constraint and diversifying selection respectively). Experimental matings between *M. smegmatis* from different genetic backgrounds would also show whether or not the locations of these cold spots are conserved or specific to the donor and recipient strains, mc^2^155 and mc^2^874.

We used BRATNextGen to characterize recombinant regions in *M. canettii*. Qualitative evidence of LGT was found previously in an alignment of these strains using the PHI test (Supply et al. 2013). Unusually high densities of SNPs in some regions of the alignment were reported as further evidence of recombination. Our analysis with BRATNextGen identified these SNP dense regions as recombinant and found additional putative recombinant regions.

Similar to *M. smegmatis*, recombinant fragments are distributed throughout the *M. canettii* genome (fig. 3); none of them is associated with MGE. Seven of the nine *M. canettii* strains had extensive recombination, with 119–232 LGT fragments/strain affecting 6–13% of the genome. STB-K and STB-J are the most distantly related *M. canettii* strains in the sample; genetic distances from these strains are approximately twice the distances between other strains (Supply et al. 2013). STB-K and STB-J had little evidence of recombination (4–5 fragments affecting 0.02–0.06% of sites). Seventy-six percent of recombinant fragments in the *M. canettii* alignment matched most closely with STB-K and STB-J, suggesting that they originated from bacteria in these or related lineages. The progeny of DCT matings in *M. smegmatis* has the recipient phenotype in the majority (90%) of cases (Wang et al. 2005); in the absence of other mechanisms of LGT, we would expect donor lineages to evolve clonally, in isolation from recipient lineages. Taken together, our observations suggest that gene flow has occurred from STB-K and STB-J lineages to the other lineages in the sample; this unidirectionality would result if STB-J/STB-K had the DCT donor phenotype and the other *M. canettii* lineages had the DCT recipient phenotype.

Recombination among *M. canettii* showed evidence of both hot spots and cold spots. The pattern of positive spatial bias was similar to *M. smegmatis*, and more modest than for the other species of bacteria in our sample (table 4). This sample is representative of all available WGS for *M. canettii*, and more genomes would allow for analysis of hot spots and cold spots at a higher resolution. Additionally, it would be interesting to compare the regions of high and low recombination between *M. canettii* and *M. smegmatis* to determine whether these patterns are conserved across mycobacteria.

Based on patterns of spatial bias, proportion of alignment affected by LGT, directionality, and absence of association with MGE, we conclude that DCT is likely the predominant mechanism of LGT among *M. canettii*. *Mycobacterium canettii* encodes the genetic elements known to be required for DCT: ESX-1, *tad*, and *lpqM* (Flint et al. 2004; Coros et al. 2008; Nguyen et al. 2009), making this mechanism plausible. Natural transformation has been observed in *M. smegmatis* and *M. avium* (Tsukamura et al. 1960; Norgard and Imaeda 1978) and could be an alternative mechanism of LGT in *M. canettii*. Little is known about the underlying mechanism or genetic requirements for natural transformation in mycobacteria. Our comparison of the genomic signatures of LGT in *M. canettii* with those of naturally transformable bacteria suggests that natural transformation is unlikely to be the sole mechanism of LGT among strains of *M. canettii*.

The *M. canettii* sample is representative of the most genetically distant isolates available rather than a single clone (Supply et al. 2013). Recombinant fragments in *M. canettii* are smaller (mean 3,291 bp) than those found in *M. smegmatis* (33 kb), but larger than those found in similarly diverse samples of *Sta. aureus* (331 bp), *Str. pneumoniae* (175 bp), and *E. faecium* (1,972 bp). An estimate of the size of transferred sequence in *Helicobacter pylori*, another naturally competent species, had a mean of 1,300 bp, also smaller than *M. canettii* (Lin et al. 2009).

We hypothesize that the discrepancy in recombinant fragment sizes between *M. smegmatis* and *M. canettii* is due to sampling differences*.* It is likely that recombinant fragments detected in the *M. canettii* sample result from multiple events in the past, and the signal of these events may have been interrupted by mutation and genomic rearrangements.

Our evaluation of DCT from WGS data has several limitations. The *M. smegmatis* data set is from experimental transconjugants rather than a natural population. This may have affected the performance of the recombination detection methods. However, BRATNextGen performed well despite violating the assumption of a natural population sample. The number of genomes available for *M. smegmatis* transconjugants and *M. canettii* is limited compared with the other species in our analyses. Additionally, the data sets are representative of different types of samples, including experimental transconjugants, clonal populations, and diverse populations. Our “hot spot” analysis could be influenced by overlapping fragments resulting from a shared recombination event in the past rather than multiple hits of the same genomic region, which would exaggerate the bias toward particular genomic regions. Despite these limitations, our evidence suggests that DCT is unique in the size of recombinant fragments, proportion of the genome affected by recombination, and spatial bias of recombinant fragments compared with other LGT mechanisms; the DCT mechanism explains the patterns of recombination present in *M. canettii* genomes.

The isolates of *M. canettii* were collected from tuberculosis cases, primarily in East Africa. Tuberculosis infections are known to occasionally involve multiple *M. tuberculosis* strains (Braden et al. 2001), and the same may be true for *M. canettii* infections. This would provide an opportunity for recombination to occur within the human host. However, it has been suggested that *M. canettii* has an environmental reservoir (Fabre et al. 2004; Koeck et al. 2011). DCT is known to occur within mixed *M. smegmatis* biofilms (Nguyen et al. 2010), a likely scenario for DCT outside the laboratory. Environmental bacteria are more genetically diverse and generally have more evidence of recombination than host-associated microbes and pathogens (Vos and Didelot 2008; Willems et al. 2012; McNally et al. 2013). The presence of a free-living life stage, with its attendant opportunities for comingling of diverse strains of *M. canettii*, could explain apparently abundant LGT among *M. canettii* relative to strictly pathogenic mycobacteria.

*Mycobacterium smegmatis* and *M. canettii* are distantly related; the identification of DCT among *M. canettii* suggests that the trait could be widespread among mycobacteria (assuming it arose once). BLAST analysis of regions unique to *M. canettii* strains did not uncover recombination between *M. canettii* and other mycobacterial species beyond those known to be shared with *M. tuberculosis* (Supply et al. 2013). This could indicate that DCT between different species of mycobacteria is uncommon. Given the requirement for extensive homologous recombination, this is plausible. Alternatively, this could simply be due to undersampling, as there are few WGS data available from *M. canettii* and environmental mycobacteria.

It has been proposed that *M. tuberculosis* arose from a mycobacterial population similar to *M. canettii* (Supply et al. 2013). There is little evidence of recombination among globally extant *M. tuberculosis* (Supply et al. 2003; Comas et al. 2013; Pepperell et al. 2013). However, the evidence of DCT among *M. canettii* suggests that DCT may have been an important mechanism for past evolution in *M. tuberculosis* as well. Regions of shared SNPs between *M. tuberculosis* and *M. canettii* strains were reported previously (Supply et al. 2013). However, we did not include *M. tuberculosis* in our BRATNextGen analysis and, therefore, did not uncover any additional recombination between these two species. In addition to mating experiments to confirm DCT between *M. canettii* strains, further inquiry into the effects of DCT on evolution and phylogenetics of *M. canettii* and other mycobacteria is warranted.

## Supplementary Material

Supplementary figures S1–S5 and tables S1 and S2 are available at *Genome Biology and Evolution* online (http://www.gbe.oxfordjournals.org/).

Supplementary Data

Supplementary Data

## References

[evu175-B1] Altschul SF, Gish W, Miller W, Myers EW, Lipman DJ (1990). Basic local alignment search tool. J Mol Biol..

[evu175-B2] Auton A, McVean G (2007). Recombination rate estimation in the presence of hotspots. Genome Res..

[evu175-B3] Braden CR (2001). Simultaneous infection with multiple strains of *Mycobacterium tuberculosis*. Clin Infect Dis..

[evu175-B4] Brosch R (2002). A new evolutionary scenario for the *Mycobacterium tuberculosis* complex. Proc Natl Acad Sci U S A..

[evu175-B5] Bruen TC, Philippe H, Bryant D (2006). A simple and robust statistical test for detecting the presence of recombination. Genetics.

[evu175-B6] Castillo-Ramírez S (2012). Phylogeographic variation in recombination rates within a global clone of methicillin-resistant *Staphylococcus aureus*. Genome Biol..

[evu175-B7] Comas I (2013). Out-of-Africa migration and Neolithic coexpansion of *Mycobacterium tuberculosis* with modern humans. Nat Genet..

[evu175-B8] Coros A, Callahan B, Battaglioli E, Derbyshire KM (2008). The specialized secretory apparatus ESX-1 is essential for DNA transfer in *Mycobacterium smegmatis*. Mol Microbiol..

[evu175-B9] Croucher NJ (2011). Rapid pneumococcal evolution in response to clinical interventions. Science.

[evu175-B10] Darling AE, Mau B, Perna NT (2010). progressiveMauve: multiple genome alignment with gene gain, loss and rearrangement. PLoS One.

[evu175-B11] de Been M, van Schaik W, Cheng L, Corander J, Willems RJ (2013). Recent recombination events in the core genome are associated with adaptive evolution in *Enterococcus faecium*. Genome Biol Evol..

[evu175-B12] DePristo MA (2011). A framework for variation discovery and genotyping using next-generation DNA sequencing data. Nat Genet..

[evu175-B13] Didelot X, Falush D (2007). Inference of bacterial microevolution using multilocus sequence data. Genetics.

[evu175-B14] Didelot X, Lawson D, Darling A, Falush D (2010). Inference of homologous recombination in bacteria using whole-genome sequences. Genetics.

[evu175-B15] Engel GA (2013). Zoonotic simian foamy virus in Bangladesh reflects diverse patterns of transmission and co-infection. Emerg Microbes Infect..

[evu175-B16] Fabre M (2004). High genetic diversity revealed by variable-number tandem repeat genotyping and analysis of hsp65 gene polymorphism in a large collection of “*Mycobacterium canettii*” strains indicates that the *M. tuberculosis* complex is a recently emerged clone of “*M. canettii*.”. J Clin Microbiol..

[evu175-B17] Fang F, Ding J, Minin VN, Suchard MA, Dorman KS (2007). cBrother: relaxing parental tree assumptions for Bayesian recombination detection. Bioinformatics.

[evu175-B18] Flint JL, Kowalski JC, Karnati PK, Derbyshire KM (2004). The RD1 virulence locus of *Mycobacterium tuberculosis* regulates DNA transfer in *Mycobacterium smegmatis*. Proc Natl Acad Sci U S A..

[evu175-B19] Gogarten JP, Townsend JP (2005). Horizontal gene transfer, genome innovation and evolution. Nat Rev Microbiol..

[evu175-B20] Gray TA, Krywy JA, Harold J, Palumbo MJ, Derbyshire KM (2013). Distributive conjugal transfer in mycobacteria generates progeny with meiotic-like genome-wide mosaicism, allowing mapping of a mating identity locus. PLoS Biol..

[evu175-B21] Gutierrez MC (2005). Ancient origin and gene mosaicism of the progenitor of *Mycobacterium tuberculosis*. PLoS Pathog..

[evu175-B22] Huson DH, Bryant D (2006). Application of phylogenetic networks in evolutionary studies. Mol Biol Evol..

[evu175-B23] Koeck J-L (2011). Clinical characteristics of the smooth tubercle bacilli “*Mycobacterium canettii*” infection suggest the existence of an environmental reservoir. Clin Microbiol Infect..

[evu175-B24] Krzywinska E, Krzywinski J, Schorey JS (2004). Naturally occurring horizontal gene transfer and homologous recombination in *Mycobacterium*. Microbiology.

[evu175-B25] Krzywinski M (2009). Circos: an information aesthetic for comparative genomics. Genome Res..

[evu175-B26] Lebreton F (2013). Emergence of epidemic multidrug-resistant *Enterococcus faecium* from animal and commensal strains. MBio.

[evu175-B27] Li H, Durbin R (2009). Fast and accurate short read alignment with Burrows-Wheeler transform. Bioinformatics.

[evu175-B28] Lin EA (2009). Natural transformation of *Helicobacter pylori* involves the integration of short DNA fragments interrupted by gaps of variable size. PLoS Pathog..

[evu175-B29] Marri PR, Bannantine JP, Paustian ML, Golding GB (2006). Lateral gene transfer in *Mycobacterium avium* subspecies *paratuberculosis*. Can J Microbiol..

[evu175-B30] Martinez-Garcia M (2012). High-throughput single-cell sequencing identifies photoheterotrophs and chemoautotrophs in freshwater bacterioplankton. ISME J..

[evu175-B31] Marttinen P (2012). Detection of recombination events in bacterial genomes from large population samples. Nucleic Acids Res..

[evu175-B32] McNally A, Cheng L, Harris SR, Corander J (2013). The evolutionary path to extraintestinal pathogenic, drug-resistant *Escherichia coli* is marked by drastic reduction in detectable recombination within the core genome. Genome Biol Evol..

[evu175-B33] McVean G, Awadalla P, Fearnhead P (2002). A coalescent-based method for detecting and estimating recombination from gene sequences. Genetics.

[evu175-B34] Minin VN, Dorman KS, Fang F, Suchard MA (2005). Dual multiple change-point model leads to more accurate recombination detection. Bioinformatics.

[evu175-B35] Mostowy R (2014). Heterogeneity in the frequency and characteristics of homologous recombination in pneumococcal evolution. PLoS Genet..

[evu175-B36] Nakamura Y, Itoh T, Matsuda H, Gojobori T (2004). Biased biological functions of horizontally transferred genes in prokaryotic genomes. Nat Genet..

[evu175-B37] Namouchi A, Didelot X, Schöck U, Gicquel B, Rocha EPC (2012). After the bottleneck: genome-wide diversification of the *Mycobacterium tuberculosis* complex by mutation, recombination, and natural selection. Genome Res..

[evu175-B38] Nguyen KT, Piastro K, Derbyshire KM (2009). LpqM, a mycobacterial lipoprotein-metalloproteinase, is required for conjugal DNA transfer in *Mycobacterium smegmatis*. J Bacteriol..

[evu175-B39] Nguyen KT, Piastro K, Gray TA, Derbyshire KM (2010). Mycobacterial biofilms facilitate horizontal DNA transfer between strains of *Mycobacterium smegmatis*. J Bacteriol..

[evu175-B40] Norgard MV, Imaeda T (1978). Physiological factors involved in the transformation of *Mycobacterium smegmatis*. J Bacteriol..

[evu175-B41] Pepperell CS (2013). The role of selection in shaping diversity of natural *M. tuberculosis* populations. PLoS Pathog..

[evu175-B42] Petes TD (2001). Meiotic recombination hot spots and cold spots. Nat Rev Genet..

[evu175-B43] Ripoll F (2009). Non mycobacterial virulence genes in the genome of the emerging pathogen *Mycobacterium abscessus*. PLoS One.

[evu175-B44] Ronquist F, Huelsenbeck JP (2003). MrBayes 3: Bayesian phylogenetic inference under mixed models. Bioinformatics.

[evu175-B45] Sawyer S (1989). Statistical tests for detecting gene conversion. Mol Biol Evol..

[evu175-B46] Supply P (2003). Linkage disequilibrium between minisatellite loci supports clonal evolution of *Mycobacterium tuberculosis* in a high tuberculosis incidence area. Mol Microbiol..

[evu175-B47] Supply P (2013). Genomic analysis of smooth tubercle bacilli provides insights into ancestry and pathoadaptation of *Mycobacterium tuberculosis*. Nat Genet..

[evu175-B48] Takeuchi N, Kaneko K, Koonin EV (2014). Horizontal gene transfer can rescue prokaryotes from Muller’s ratchet: benefit of DNA from dead cells and population subdivision. G3.

[evu175-B49] Thomas CM, Nielsen KM (2005). Mechanisms of, and barriers to, horizontal gene transfer between bacteria. Nat Rev Microbiol..

[evu175-B50] Tsukamura M, Hasimoto M, Noda Y (1960). Transformation of isoniazid and streptomycin resistance in *Mycobacterium avium* by the desoxyribonucleate derived from isoniazid- and streptomycin-double-resistant cultures. Am Rev Respir Dis..

[evu175-B51] Veyrier F, Pletzer D, Turenne C, Behr MA (2009). Phylogenetic detection of horizontal gene transfer during the step-wise genesis of *Mycobacterium tuberculosis*. BMC Evol Biol..

[evu175-B52] Vos M, Didelot X (2008). A comparison of homologous recombination rates in bacteria and archaea. ISME J..

[evu175-B53] Wang J, Karnati PK, Takacs CM, Kowalski JC, Derbyshire KM (2005). Chromosomal DNA transfer in *Mycobacterium smegmatis* is mechanistically different from classical Hfr chromosomal DNA transfer. Mol Microbiol..

[evu175-B54] Wang J, Parsons LM, Derbyshire KM (2003). Unconventional conjugal DNA transfer in mycobacteria. Nat Genet..

[evu175-B55] Willems RJL (2012). Restricted gene flow among hospital subpopulations of *Enterococcus faecium*. MBio.

[evu175-B56] Yahara K (2012). Genome-wide survey of mutual homologous recombination in a highly sexual bacterial species. Genome Biol Evol..

[evu175-B57] Zimin A (2013). The MaSuRCA genome Assembler. Bioinformatics.

