# Supplementary material for: Genomic Signatures of Distributive Conjugal Transfer among Mycobacteria
Source: Genome Biol Evol. 2014 Aug 30;6(9):2489–500. doi: 10.1093/gbe/evu175 (PMC4202316; doi:10.1093/gbe/evu175)
Supplement: Supplementary Data [file supp_evu175_genomicSignaturesDCT_052014_supplement.pdf]

Supplementary Figure Legends:

Figure S1: Likelihood surface of $2N_er$ from LDhat. The population recombination rate (ρ) for *M. smegmatis* was estimated using the pairwise program implemented in LDhat ((Auton and McVean 2007)). ρ is shown on the X axis , and the likelihood is shown on the Y axis. The plot was made from summarise.pairwise R script found at http://ldhat.sourceforge.net/R/coalescent.r. The data are consistent with a wide range of recombination rates in these analyses.

Figure S2: Size distributions of gaps between recombinant fragments in *M. smegmatis*. Sizes of correct gaps from true breakpoint data from Gray et al. 2013 are shown in dark gray. Sizes of incorrect gaps from BRATNextGen analysis, defined as gaps where the number of SNPs compared to the recipient genome were equal between the donor and transconjugants, are shown in light gray. The vertical line depicts the cutoff of 475 bp (< 5% of correct gaps) chosen to close gaps in BRATNextGen data. This cutoff maximizes the number of incorrect gaps that are closed while minimizing the number of correct gaps closed.

Figure S3: Size distribution of fragments in *M. smegmatis* from experimental data and BRATNextGen. Medians for each distribution are marked with a red line. *M. smegmatis* (Experimental) shows the size distribution of recombinant fragments identified by Gray et al. 2013. *M. smegmatis* (BNG) shows the distribution from BRATNextGen analysis of the same sample of *M. smegmatis*. *M. smegmatis* (BNG Corrected) shows the distribution from BRATNextGen with gaps less than 475 bp closed. The X axis shows the size of recombinant fragments in basepairs on a log scale. The Y axis shows the frequency of fragment sizes in each dataset. Closing gaps less than 475 bp moves the distribution of fragment size from BRATNextGen closer to the true distribution.

Figure S4: Recombinant fragments identified with BRATNextGen. Recombinant tracts identified with BRATNextGen are shown in black . Each circle corresponds to one strain of bacteria. Plot made with Circos (Krzywinski et al. 2009). A) *Streptococcus pneumoniae* B) *Staphylococcus aureus* C) *Enterococcus faecium*

Figure S5: Phylogenetic tree of *Mycobacterium canettii*. We used MrBayes v3.2.1 (Ronquist and Huelsenbeck 2003) to infer a Bayesian phylogeny from an alignment of *M. canettii* SNPs with repetitive regions and gaps removed . We used a General Time Reversible (GTR) model with gamma-distributed rates in this analysis. The MCMC was 1,000,000 generations sampled every 100 generations with 25% of samples discarded as burn-in. Tree visualization created with FigTree v1.4.0 (http://tree.bio.ed.ac.uk/software/figtree/).

Table S1: Accession numbers for whole genome sequence data

Mycobacterium smegmatis [a]	ERP002619
Mycobacterium canettii [a,b]	HE572590, PRJEB94/FO203507, PRJEB93/FO203508, PRJEB92/FO203509, PRJEB95/FO203510, PRJEB584/CAOL00000000, PRJEB585/CAOM00000000, PRJEB586/CAON00000000, PRJEB587/CAOO00000000
Streptococcus pneumoniae	BRATNextGen Results from (Marttinen et al. 2012)
Staphylococcus aureus	BRATNextGen Results from (Castillo-Ramírez et al. 2012)
Enterococcus faecium	BRATNextGen Results from (Been et al. 2013)
Streptococcus pneumoniae Global Dataset [c]	NC_011900, NC_011072, NC_008533, NC_012467, NC_021006, NC_014251, NZ_CM001835, NC_012468, NC_012469, NC_003098, NC_010380, NC_017593, NC_017592, NC_017591, NC_014498, NC_021026, NC_003028, NC_012466, NC_022655, NC_010582, NC_014494
Staphylococcus aureus Global Dataset [c]	NC_017331, NC_013450, NC_018608, NC_007793, NC_022113, NC_007795, NC_003923, NC_017763, NC_021670, NC_002951, NC_002952, NC_020529, NC_017343, NC_017340, NC_021554, NC_022442, NC_022226, NC_022222, NC_017673, NC_002745, NC_021059, NC_007622, NC_017351, NC_017337, NC_017333

[a] European Nucleotide Archive

[b] NCBI GenBank

[c] NCBI Reference Sequence

Table S2: Proportion of strains with greater than 5% of genome identified as recombinant.

Species	Proportion
Mycobacterium smegmatis	0.67
Mycobacterium canettii	0.78
Enterococcus faecium	0.22
Streptococcus pneumoniae	0.19
Staphylococcus aureus	0.0

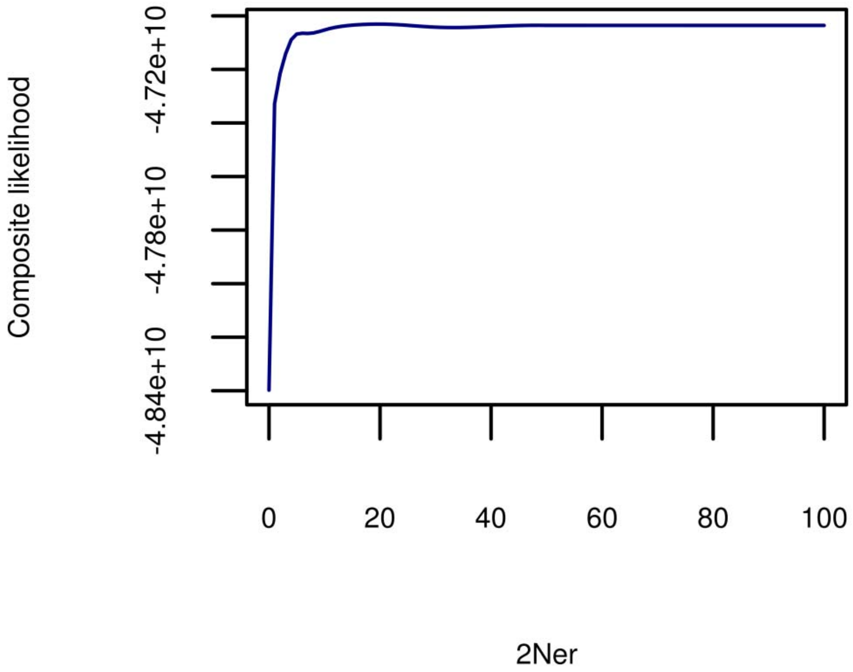
Composite likelihood
-4.72e+10
-4.78e+10
-4.84e+10
0
20
40
60
80
100
2Ner

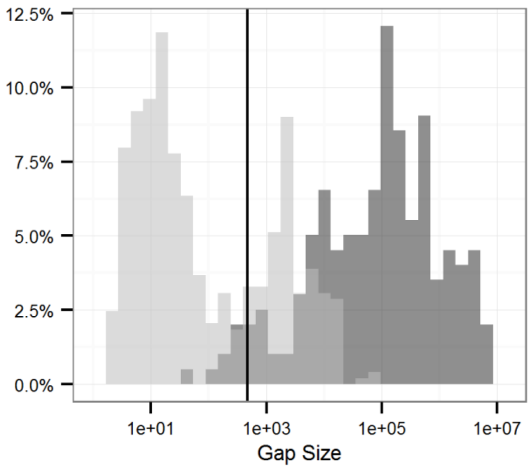
12.5%
10.0%
7.5%
5.0%
2.5%
0.0%
1e+01
1e+03
1e+05
1e+07
Gap Size

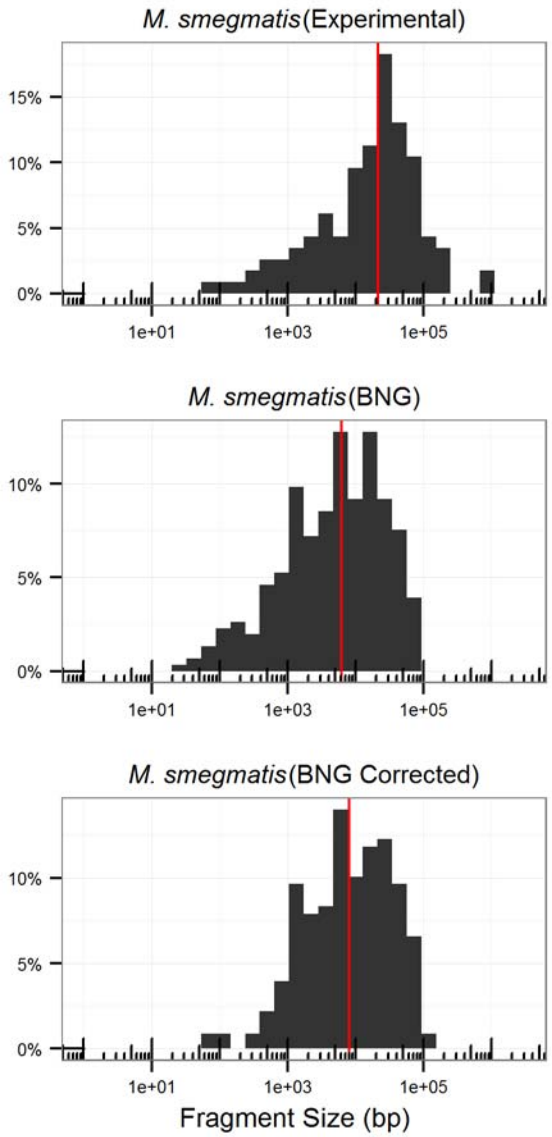
M. smegmatis (Experimental)
15%
10%
5%
0%
1e+01
1e+03
1e+05
M. smegmatis (BNG)
10%
5%
0%
1e+01
1e+03
1e+05
M. smegmatis (BNG Corrected)
10%
5%
0%
1e+01
1e+03
1e+05
Fragment Size (bp)

A

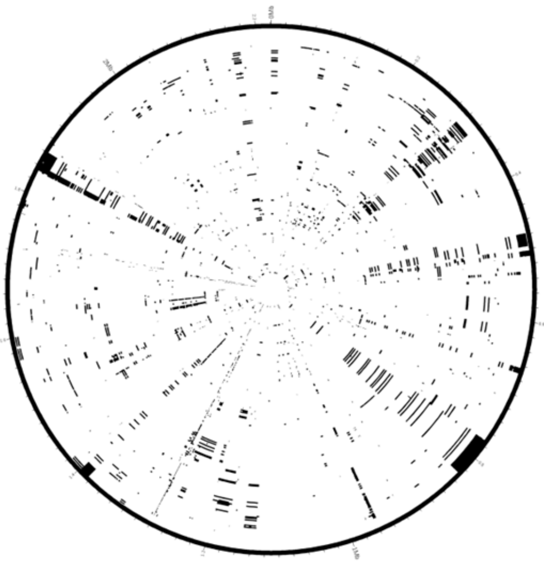

B

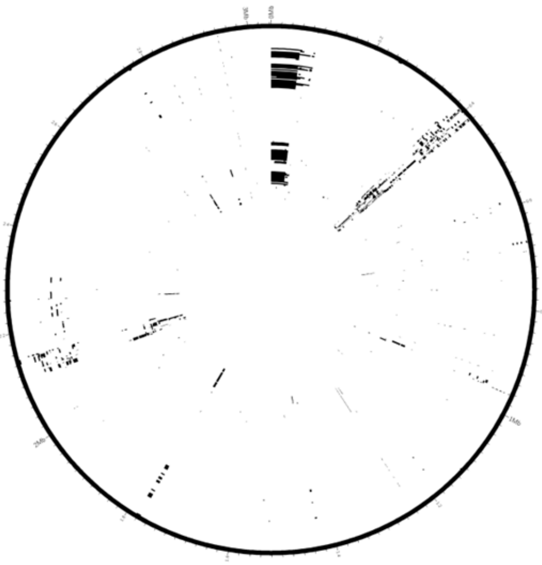

C

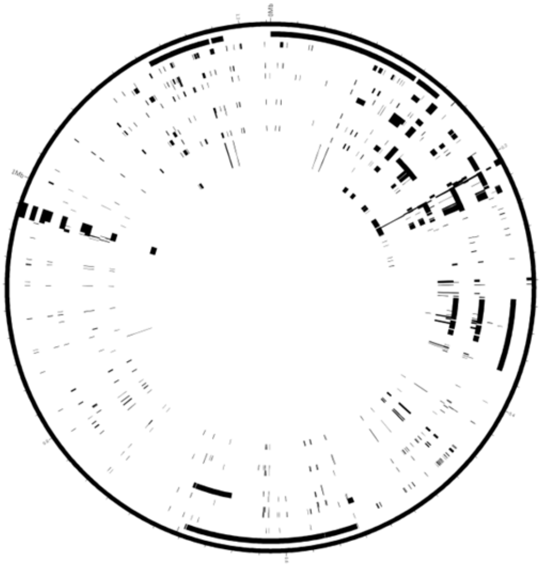

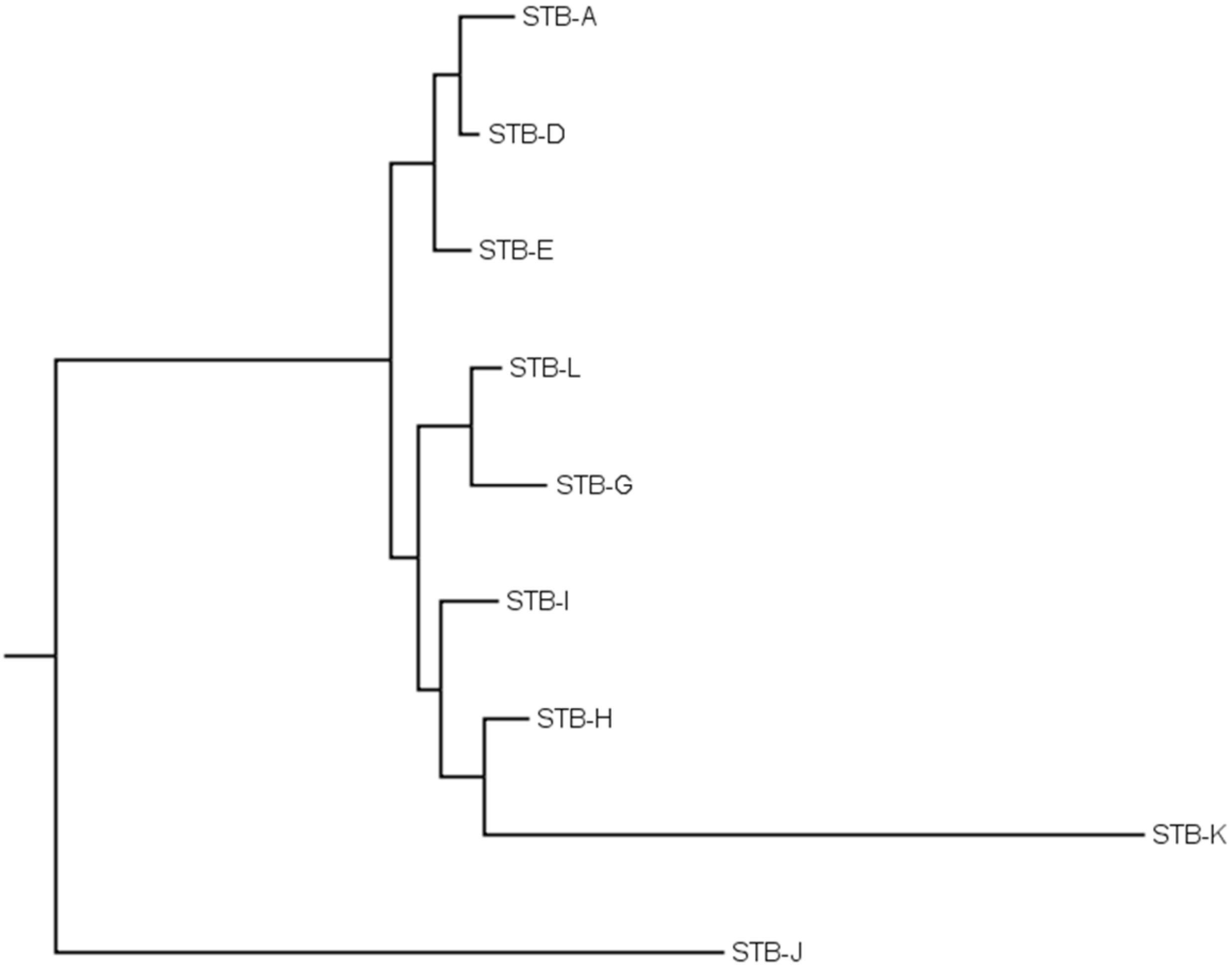
STB-A
STB-D
STB-E
STB-L
STB-G
STB-I
STB-H
STB-K
STB-J
