# Supplementary material for: Genomic Signatures of Distributive Conjugal Transfer among Mycobacteria
Source: Genome Biol Evol. 2014 Aug 30;6(9):2489–500. doi: 10.1093/gbe/evu175 (PMC4202316; doi:10.1093/gbe/evu175)
Supplement: Supplementary Data [file supp_6_9_2489_v2_index.html]

Genomic Signatures of Distributive Conjugal Transfer among Mycobacteria — Supplementary Data 

# Genomic Signatures of Distributive Conjugal Transfer among Mycobacteria

## Supplementary Data

files

**Files in this Data Supplement:**

- Supplementary Data - pdf file
